# Functional outcomes after anterior cruciate ligament reconstruction: unravelling the role of time between injury and surgery, time since reconstruction, age, gender, pain, graft type, and concomitant injuries

**DOI:** 10.1186/s13102-023-00663-x

**Published:** 2023-04-01

**Authors:** Daniel Niederer, Michael Behringer, Thomas Stein

**Affiliations:** 1 Institute of Occupational, Social and Environmental Medicine, Goethe University Frankfurt, Frankfurt, Germany; 2 Department of Sports Medicine and Exercise Physiology, Goethe University Frankfurt, Ginnheimer Landstraße 39, 40487 Frankfurt, Germany; 3 Sporthologicum Frankfurt - Center for Sport and Joint Injuries, Frankfurt, Germany

**Keywords:** Return to sport, Return to play, Graft type, Rehabilitation, Functional test, Re-injury

## Abstract

**Background:**

Numerous individual, temporal, injury- and surgery-specific factors impact the functional capacity during rehabilitation, return to sports (RTS), and re-injury prevention after an anterior cruciate ligament (ACL) reconstruction.

**Purpose:**

This multicentre cohort study evaluated the isolated and interactive contributions of time between injury and surgery, time since reconstruction, age, gender, pain, graft type, and concomitant injuries as to inertial sensor-assessed motor function after ACL reconstructions in multiple linear mixed model regressions.

**Methods:**

Anonymized data were retrieved from a nationwide German registry. In this cohort study, patients with an acute unilateral ACL rupture, with or without concomitant ipsilateral knee injuries, and having passed an arthroscopically assisted anatomic reconstruction were included. Potential predictors were age [years], gender/sex, time since reconstruction [days], time between injury and reconstruction [days], concomitant intra-articular injuries (isolated ACL tear, meniscal tear, lateral ligament, unhappy triad), graft type (hamstrings, patellar, or quadriceps tendon autograft), and pain during each measurement (visual analogue scale 0–10 cm). Repeated inertial motion unit-assessments of a comprehensive battery of classic functional RTS test were performed in the course of the rehabilitation and return to sports: Joint position sense/kinesthesia (Angle reproduction error [degrees]), Dynamic Balance Composite score [cm] of the Y-Balance test), drop jumps (Knee displacement [cm]), Vertical hop (Hopping height [mm]), Speedy jumps (Duration [seconds]), Side hops (Number of hops [n]), single leg hop for distance (hopping distance [cm]). Repeated measures multiple linear mixed models investigated the impact and nesting interaction of the potential predictors on the functional outcomes.

**Results:**

Data from 1441 persons (mean age 29.4, SD 11.8 years; 592 female, 849 male) were included. Most had an isolated ACL rupture: n = 938 (65.1%). Minor shares showed lateral ligament involvement: n = 70 (4.9%), meniscal tear: n = 414 (28.7%), or even unhappy triad: n = 15 (1%). Several predictors such as time between injury and reconstruction, time since reconstruction (estimates for n_days_ ranged from + .05 (i.e., an increase of the hopping distance of 0.05 cm per day since reconstruction occurs) for single leg hop for distance to + 0.17 for vertical hopping height; p < 0.001), age, gender, pain, graft type (patellar tendon graft: estimates between + 0.21 for Y-balance and + 0.48 for vertical hop performance; p < 0.001), and concomitant injuries contribute to the individual courses of functional abilities of the reconstructed side after ACL reconstruction. The unimpaired side was mostly influenced by sex, age, the time between injury and reconstruction (estimates between − 0.0033 (side hops) and + 0.10 (vertical hopping height), p < 0.001)), and time since reconstruction.

**Conclusions:**

Time since reconstruction, time between injury and reconstruction, age, gender, pain, graft type, and concomitant injuries are not independent but nested interrelating predictors of functional outcomes after anterior cruciate ligament reconstruction. It might not be enough to assess them isolated; the knowledge on their interactive contribution to motor function is helpful for the management of the reconstruction (earlier reconstructions should be preferred) deficit-oriented function-based rehabilitation (time- and function based rehabilitation instead of solely a time- or function based approach) and individualized return to sports strategies.

## Introduction

In comparison to the first anterior cruciate ligament (ACL)-rupture risk, the risk of suffering from a subsequent injury after (ACL) reconstruction increases at least tenfold [1, 2]. More detailed, a cumulated (ipsi- and contralateral, re-injury & graft failure) recurrence risk of 10–25% is reported in the literature [1, 2]. Such secondary injuries often occur during the return to sports (RTS) process [1].

It is a major goal of the RTS-process to lead athletes back to activity, training, and competition without exposing him/her to an excessively high risk for a subsequent rupture [3]. Important criteria to be fulfilled for an RTS release, beyond morphological graft healing and psychosocial readiness, is the restoration of neuromuscular and motor function injury [4–6]. This function is mostly assessed by a combination of simple clinical tests, dynamic strength, and hop/jump tests. Functional deficits or limb asymmetries, where the affected leg’s performance is compared to the putatively unaffected contralateral leg’s performance, seems to be predictive for a second ACL injury [4–6]. Improving or even restoring these functional abilities vice versa decreases the deficit; in this scenario, that leads to a decrease in the subsequent injury risk [7].

The biology of graft healing and maturation is of great importance during the continuum of rehabilitation, RTS, and re-injury prevention. Based on the individual development in biological healing, functional skills, and psychological readiness, the time slot before RTS is variable [8]. Although it is not possible to define fixed time points at which a certain goal or functional ability should be reached, time (both before and after the reconstruction) is, nevertheless, one factor to consider after ACL reconstructions [9]. It is, for example, likely that the graft healing will take more time than the time until RTS success [10].

Numerous graft types in ACL reconstruction are adopted. In a recently published survey, 90.4% of the interviewed surgeons preferentially used hamstring tendon autografts for most ACL reconstruction followed by bone-patellar tendon-bone grafts [11, 12]. Hamstrings tendon autograft is thus, in particular in Europe, the—by far—most used graft type. A comparably new graft type is based on quadriceps tendons. Quadriceps tendon autografts show comparable clinical and functional outcomes and graft survival rate like other autografts, but significantly less harvest site pain when compared to patella autografts [13]. Further, quadriceps tendon autografts may lead to better functional outcome scores when compared with hamstrings autograft [13].

Conclusively, a multitude of individual and spatiotemporal factors interact during the rehabilitation, RTS, and re-injury prevention processes after ACL reconstruction. Beyond those highlighted so far, age, sex/gender, pain intensity/perception during performance, and concomitant knee injuries like Meniscus and collateral ligament injuries, or even unhappy triad must also be taken into account when the neuromuscular function after ACL reconstruction is rated [14–16].

Considering the multitude of factors with the aim to derive individual courses of functional abilities and the impact of major contributors to these abilities is helpful for the for the management of deficit-oriented function-based rehabilitation strategies after ACL reconstructions [17, 18]. Aiming to provide such a more general view on the course of functional abilities after ACL reconstruction, the purpose of our cohort study was to evaluate the contribution of time between injury and surgery, time since reconstruction, age, gender, pain, graft type, and concomitant injuries as isolated and interactive contributors to inertial sensor-assessed motor function after ACL reconstructions in a multiple linear mixed model regression approach. We hypothesized that numerous of the potential contributors interact with each other in their way to interactively impact on different functional abilities.

## Methods

### Study design, ethics, and informed consent

In this multicenter cohort study, all methods were performed in accordance with the relevant guideline. Data were extracted from a data registry. The registry is the nationwide database from an enterprise (OPED GmbH, Valley, Germany). The register was initiated in January 2018, data from initiation until October 2020 were included in this analyses. As all data was retrieved completely anonymized from a registry, ethical approval is not relevant for this type of analysis.

Informed consent was obtained from all participants and (below 16 years of age) from their legal guardian. All data were assessed as a part of the functional assessment during the rehabilitation after ACL-reconstruction, no measurement or measures for study purposes were additionally undertaken.

The database consists of prospectively assessed multiple, in particular functional, measurements. More detailed, the measurements included in the study were used to assess the participants function during their formal medically prescribed standard rehabilitation process.

### Inclusion and exclusion criteria

The data from all database patients (children, adolescent, adult males, females, and diverse) with an acute unilateral ACL rupture with or without concomitant ipsilateral knee injuries (meniscal tear, lateral ligament involvement, unhappy triad) and having passed an arthroscopically applied, anatomical reconstruction was included. Main exclusion criteria were bilateral lower limb injuries, other major injuries than ACL tears with exception of secondary knee injuries, pregnancy, and severe diseases potentially affecting motor function.

Independent outcome variables.

The following potential outcome modifiers were extracted for each participant and at each of the repeated measurement: age range (0–15, 16–20, 21–25, 26–30, 31–40, 41–50, and above 50 years)), gender/sex (male, female, divers/unknown), time since reconstruction [days], time between injury and reconstruction [days], concomitant intra-articular injuries (isolated ACL tear, meniscal tear, lateral ligament, unhappy triad), graft type (hamstrings, patellar, or quadriceps tendon autograft), and pain intensity during the measurement (visual analogue scale 0–10 cm).

### Dependent outcomes: functional tests

All functional tests were performed from experienced personnel (athletic trainer, physiotherapists, sports medicine or orthopedic physicians, or sports therapists). A standard operating procedure and test manual is used to perform the standardized test battery. The functional tests display increased chaos, starting from high control angle reproduction tasks to high chaos such as speedy jump [19]. The test were selected by the same experience assessors based on standard principles of function (and time-) based rehabilitations after ACL reconstructions [18]. Details on the outcomes, the underlying function, the tool used, the conduction, and the testing criteria are displayed in Table 1.

**Table 1:** Overview of the functional outcomes. Each functional ability is described by the corresponding outcome and tool used to assess the ability, its test quality criteria, conduction and the (positive) decision criteria

Function	Outcome	Tool	Test quality criteria	Conduction
Joint position sense	Absolute angle reproduction error [degrees]	Intertial motion unit	Unknown, ICC = 0.31–0.82 (sitting) and 0.17–0.75 (prone) [21]	Start point see Flexion. A target angle in flexion RoM must be reached and reproduced after a 3 s break
Dynamic Balance	Composite score	Y-Balance test (imprinted on a carpet or affixed on the floor)	Good interrater test–retest reliability, acceptable level of measurement error [22]	Single-(test)leg standing, other leg must be moved anterior, posterior later and posterior medial as fast as possible without losing postural control. Composite score is calculated by adding all three single values divided by three times the limb length, this all multiplied by one hundred
Vertical jumps and hops measurement	Knee displacement [cm]	Drop Jump, Intertial motion unit	ICC = 0.93 [23]	Bipedal hip-width stance on a box with a 32 cm target-height. A bipedal drop jump follows: frontal step – drop – reactive jump with the shortest possible ground contact time
	Hopping height [mm]	Vertical hop, intertial motion unit	ICC = 0.89 -0.97 [24]; Average error with intertial sensors: -0.4 to 2.2 cm [25], ICC = .98	Unipedal stance on the ground, one-legged counter movement jumps
horizontal jumps and hops	Time to completion [seconds]	Speedy jump, intertial motion unit	ICC = 0.792–0.825 [26]	A small trail must be passed as fast as possible in one-legged hops
	Number of hops in 30 s [n]	Side hops intertial motion unit	Unknown	Participants have to hop the participants hop over laterally (to the foot’s respective side) over a square on the floor with 30 × 30 cm edge length with hands on their hips as fast as possible [27] (article in German)
	Hopping distance [cm]	Single leg hop for distance	The measurement properties for the SLHD are excellent, reliability is ICC = 0.97 (CI 0.9–0.99) and the standard error of measurement is 3.5% [28]	The participants stand on one leg with the toes behind the rear line of the square. He/she then hops as far as possible and has to land in a controlled manner. The whole hop must be performed one-legged

*ICC* intraclass correlation coefficient; *ACL* anterior cruciate ligament; *RTS* return to sports

### Outcomes assessment

All joint position sense, vertical jumps and hops measurement, side hops, and the speedy jump outcomes were assessed using a single inertial sensor (Orthelligent Pro, OPED GmbH, Valley, Germany). The non-invasive external three-dimensional wireless sensor was positioned at the highest circumference of the lower leg using an elastic band; the sensor itself was placed on the tibia. Using inertial sensor techniques may detect re-injury associated movements behavior more adequate than the “classic” outcomes of functional return to sport testing [29].

The sensor consists of a 9-axis MEMS MotionTracking device (TDK InvenSense, Chūō, Tokio, Japan); with 3 accelerometers (measurement range ± 2 g to ± 16 g), a 3-axis gyroscope (± 250 to ± 2000 degrees per second), and a 3-axis magnetometer. The device was zeroed prior to each measurement.

Sample rate was (accelerometer) 4.5 kHz to (Gyroscope) 9.0 kHz. The data was down-sampled (4:1) and filtered for the further analysis. A low pass and Kalman filter was applied.

The tool, all test settings and outcomes, and the setup has been validated against a gold-standard movement assessment system and was found to be valid in terms of or the objective assessment of movements of the lower limb [30].

### Statistical analysis

The statistical analyses were performed blinded to the data retrieval from the database.

Range data plausibility check was undertaken for all independent and dependent outcomes; the data were cleared accordingly.

Repeated measures linear mixed models (multilevel analysis) investigated the impact and interaction of the individual (random effects) predictors (level 2), and of time (level 1) on the functional outcomes’ values. 2-LL estimates were adopted to build the models. Due to the considerable amount of missing information on the graft type, these analyses were, partially, contributed separately. In detail, the analyses which were once modelled including and once without the graft type are highlighted with an empty row (in the tables) between the graft type and the other contributors. The size of the estimates highlight the size of the effect of the independent on the dependent variable (always in the units used), the direction is indicated by the leading sign (minus indicates a negative association).

All analyses were performed in SPSS version 25 (IBM Corporation, New York, NY, USA), an alpha-error of 5% was considered as a relevant cut-off significance value, all p-values below are interpreted as statistically significant.

## Results

The entries from 1629 persons were screened. Data from 1441 individuals were included into the final analysis. Exclusion reasons for the others were: duplicate entries (n = 48), critical data (identity for repeated measures) missing (n = 84), non-ACL-tear (n = 56). For those included, graft type distributions was as follows (always displayed as absolute numbers and percentage share): hamstrings (semitendinosus with or without gracilis tendon): n = 566 (39.3%); patellar tendon graft: n = 40 (2.8%); quadriceps tendon graft: n = 139 (9.6%); unknown: n = 696 (48.3%). Secondary injuries /issues distribution was: isolated ACL rupture: n = 938 (65.1%); lateral ligament (Ligamentum collaterale tibiale) involvement: n = 70 (4.9%); meniscal tear: n = 414 (28.7%); unhappy triad (Combined ACL, Meniscus medialis, and Ligamentum collaterale tibiale tea): n = 15 (1%); unknown: 4 (0.3%).

The samples’ mean age was 29.4 years (standard deviation 11.8 years), 592 females and 849 males with a mean body mass index of 24.8 kg/m^2^ (standard deviation 3.5 kg/m^2^) were analyzed. Mean time passed between the injury and the reconstruction was: median 50 days, mean 135 days (standard deviation 193 days).

**Fig. 1:**
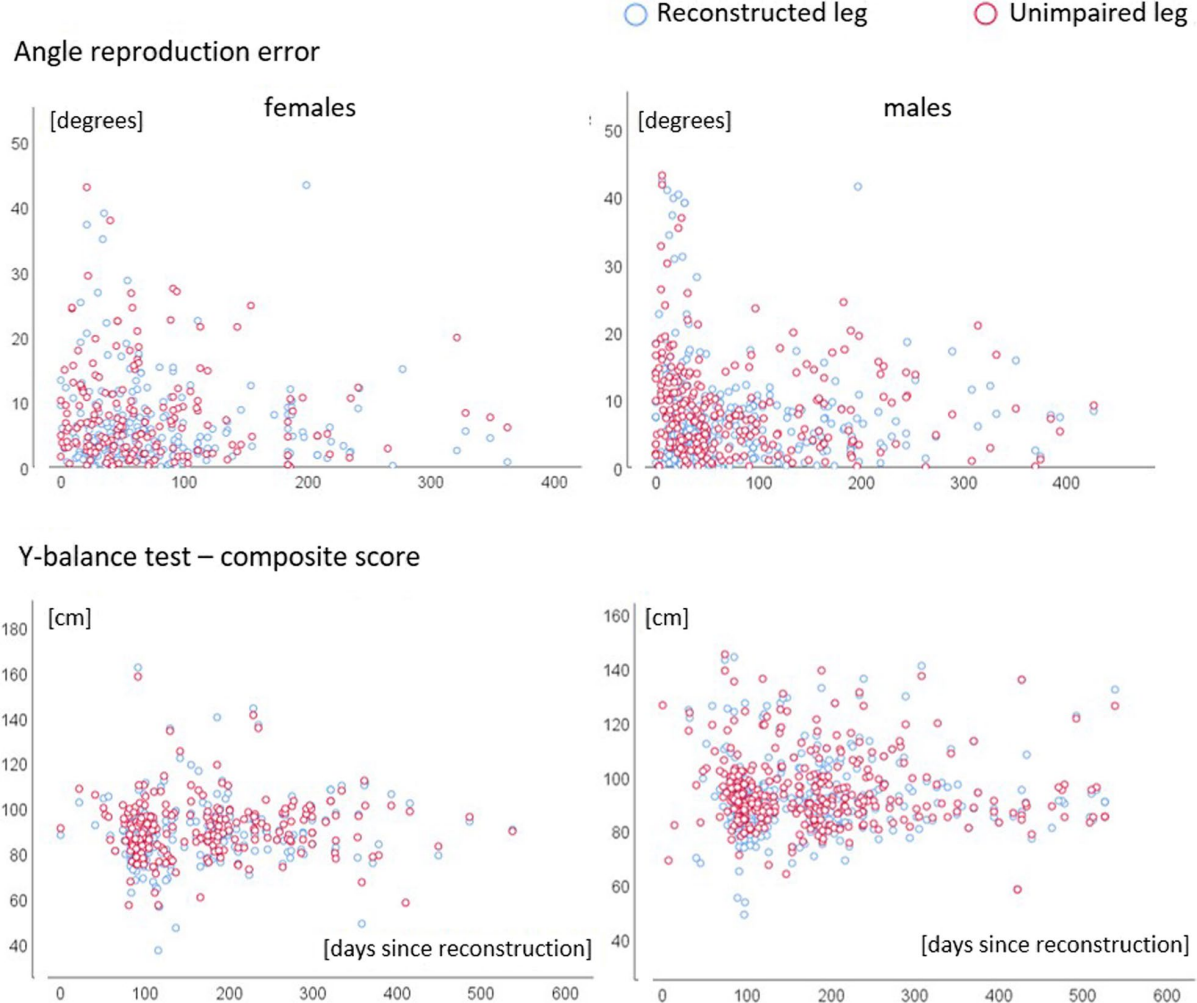
Scatterplots for kinesthesia (angle reproduction, above) and postural balance (y-balance test, below). Individual data (dots) for the days since reconstruction (x-axis) and the values of the outcomes (y-axis), separated for females and males, are displayed

Figure 1 displays the individual data for the kinesthesia/joint position sense and postural balance measurements (angle reproduction and y-balance test). The corresponding analysis’ outcomes are highlighted in Table 2. Mean pain intensity during measurement was 0.51 points (standard deviation 1.32 points) for the reconstructed side. Variance explained by interindividual differences was 7% (angle reproduction), and 29% (Y-Balance).

**Table 2:** Estimates, confidence intervals, and statistical outcomes for the mixed models for s kinesthesia and balance (A: kinesthesia, angle reproduction error, B: postural balance, Y-Balance composite score); both times separated by the reconstructed and unimpaired leg

		ACL reconstructed side					Contralateral side				
		Estimate	Standard error	95% Confidence interval		p value	Estimate	Standard error	95% Confidence interval		p value
				Lower level	Upper level				Lower level	Upper level	
*A Angle reproduction error [degrees] n = 368 individuals, n = 2196 measurements*											
	Constant	4.2	7.1	− 9.8	18.1	0.56	10.85	7.03	− 3.01	24.72	0.12
Time	Time from injury to reconstruction [days]	− 0.0008	0.0004	− 0.0015	− 0.0002	**0.02**	0.002	0.002	− 0.001	0.005	0.27
	Time since reconstruction [days]	− 0.001	0.005	− 0.01	0.01	0.90	− 0.01	0.01	− 0.02	0.01	0.33
Individual characteristics	Being female	− 1.1	0.7	− 2.4	0.2	0.09	− 1.10	0.96	− 2.99	0.79	0.25
	Being male (ref.)	0	0				0	0			
	Pain intensity [a. u.]	0.78	0.24	0.32	1.251	**0.001**	0.00	0.00			
Injury patterns	Isolate ACL rupture	3.0	7.1	− 10.8	16.9	0.67	− 2.00	6.99	− 15.78	11.78	0.77
	Lateral ligament involved	16.9	7.6	2.1	31.8	**0.03**	3.07	8.03	− 12.75	18.89	0.70
	Meniscal tear	2.9	7.1	− 11.0	16.9	0.68	− 1.8	7.0	− 15.7	12.0	0.80
	Unhappy triad (ref.)	0.0	0.0				0.0	0.0			
*B* *Y-Balance composite score [cm]* *n = 559 individuals, n = 1540 measurements*											
	Constant	94.62	4.36	86.020	103.22	**0.001**	94.211	5.70	82.92	105.502	**0.001**
Time	Time from injury to reconstruction [days]	− 0.004	0.007	− 0.018	0.010	0.6	− 0.002	0.008	− 0.018	0.015	0.8
	Time since reconstruction [days]	0.001	0.002	− 0.003	0.006	1	0.00	0.003	− 0.005	0.005	1
Individual characteristics	Being female	− 4.49	2.397	− 9.22	0.23	0.062	− 6.24	3.24	− 12.660	0.19	0.06
	Being male (ref.)	0	0				0	0			
	Age [years]	− 0.1	0.1	− 0.37	0.1	0.3	− 0.22	0.2	− 0.5	0.10	0.2
	Pain intensity [a. u.]	− 2.5	0.9	− 4.38	− 0.7	**0.001**	0.00	0.0			
Graft type	Hamstrings tendon	7.0	3.5	0.16	13.8	**0.001**	8.44	4.6	− 0.7	17.60	0.1
	Patellar tendon	21.78	8.30	5.415	38.14	**0.01**	17.231	13.44	− 9.37	43.835	0.20
	Quadriceps tendon (ref.)	0	0				0	0			

*N* numbers; *ACL* anterior cruciate ligament; *LL* lower level; *UL* upper level (of the 95% confidence interval)

**Fig. 2:**
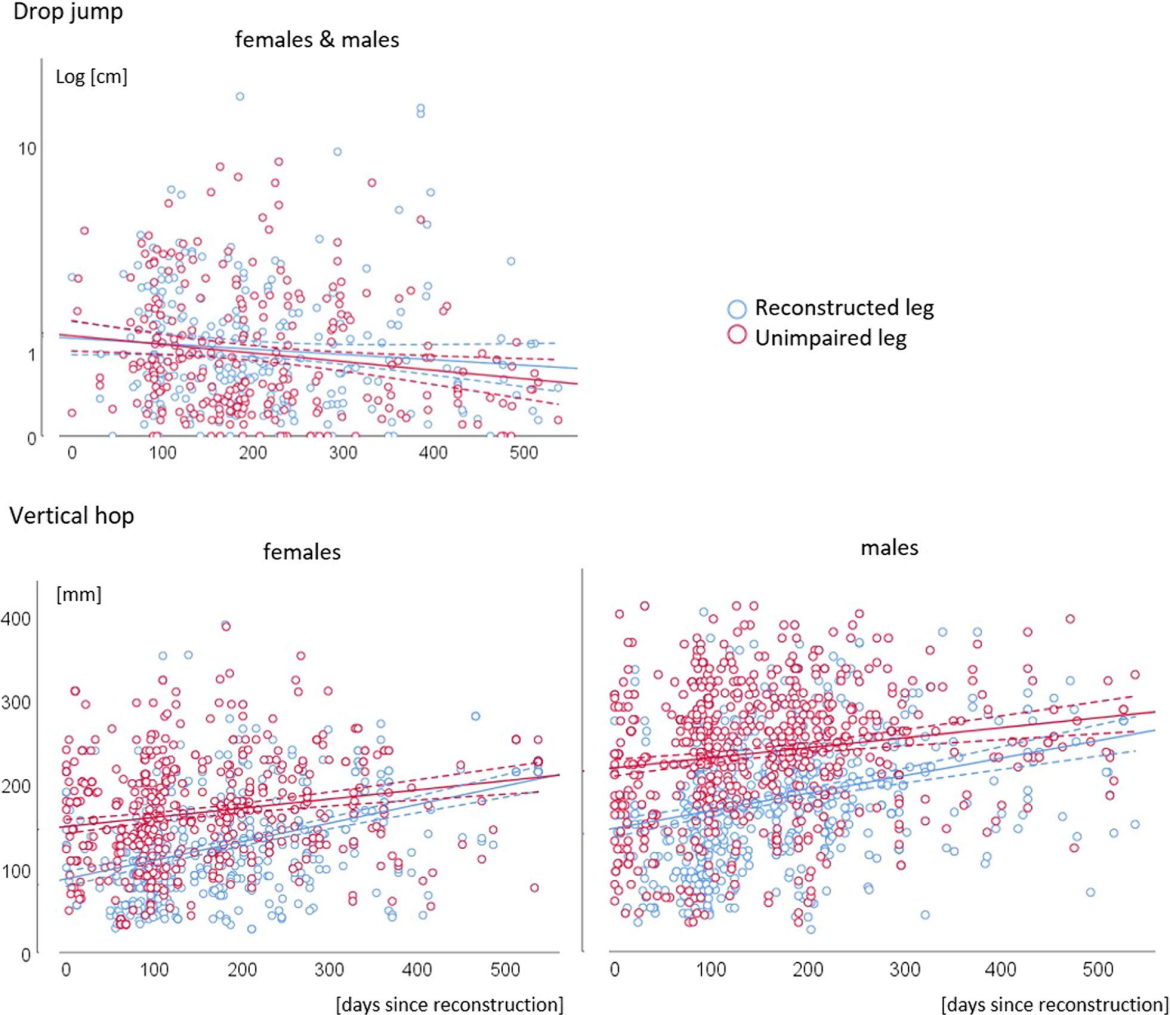
scatterplot for the vertical jump and hop measurement drop jump (above) and vertical hop (below). Individual data (dots) for the days since reconstruction (x-axis) and the values of the outcomes (y-axis), and the corresponding regression curves are displayed

Figure 2 displays the individual data for the vertical jump and hop measurement (Drop jump and vertical hop). The corresponding analysis’ outcomes are highlighted in Table 3. Mean pain intensity during measurement was 0.36 points (standard deviation 1.05 points). Variance are explained by interindividual differences was 29% (Drop jumps), and 3% (Vertical hop).

**Table 3:** Estimates, confidence intervals, and statistical outcomes for the mixed models for the vertical jump and hop measurements: drop jump (above) and vertical hop (below); both times separated by the reconstructed and unimpaired leg

		ACL reconstructed side					Contralateral side				
		Estimate	Standard error	95% Confidence interval		p value	estimate	Standard error	95% Confidence interval		p value
				Lower level	Upper level				Lower level	Upper level	
*A Drop Jump knee displacement [cm] n = 356 individuals, n = 939 measurements*											
	Constant	1.40	0.14	1.13	1.67	**0.001**	1.21	0.10	1.00	1.41	**0.001**
Time	Time since reconstruction [days]	0.00005	0.00023	− 0.00040	0.00050	0.83	0.0004	0.0001	0.0001	0.0007	**0.01**
*B Vertical hopping height [mm] n = 1010 individuals, n = 4823 measurements*											
	Constant	207.2	9.4	188.7	225.6	**0.001**	269.8	8.7	252.7	286.9	**0.001**
Time	Time from injury to reconstruction [days]	0.17	0.02	0.13	0.21	**0.001**	0.10	0.02	0.06	0.14	**0.001**
	Time since reconstruction [days]	− 1.9	0.3	− 2.4	− 1.4	**0.001**	− 1.9	0.2	− 2.3	− 1.4	**0.001**
Individual characteristics	Age [years]	− 0.017	0.005	− 0.028	− 0.006	**0.001**	− 0.011	0.003	− 0.017	− 0.005	**0.001**
	Being female	− 44.7	5.1	− 54.7	− 34.8	**0.001**	− 51.0	5.0	− 60.7	− 41.2	**0.001**
	Being male (ref.)	0	0				0	0			
Graft type	Hamstrings tendon	11.0	13.0	− 14.5	36.6	0.4	− 16.8	13.2	− 42.9	9.2	0.2
	Patellar tendon	47.5	22.6	2.9	92.0	**0.001**	16.8	25.1	− 32.6	66.3	0.5
	Quadriceps tendon (ref.)	0	0				0	0			

*N* numbers; *ACL* anterior cruciate ligament; *LL* lower level; *UL* upper level (of the 95% confidence interval); *ref.* reference

**Fig. 3:**
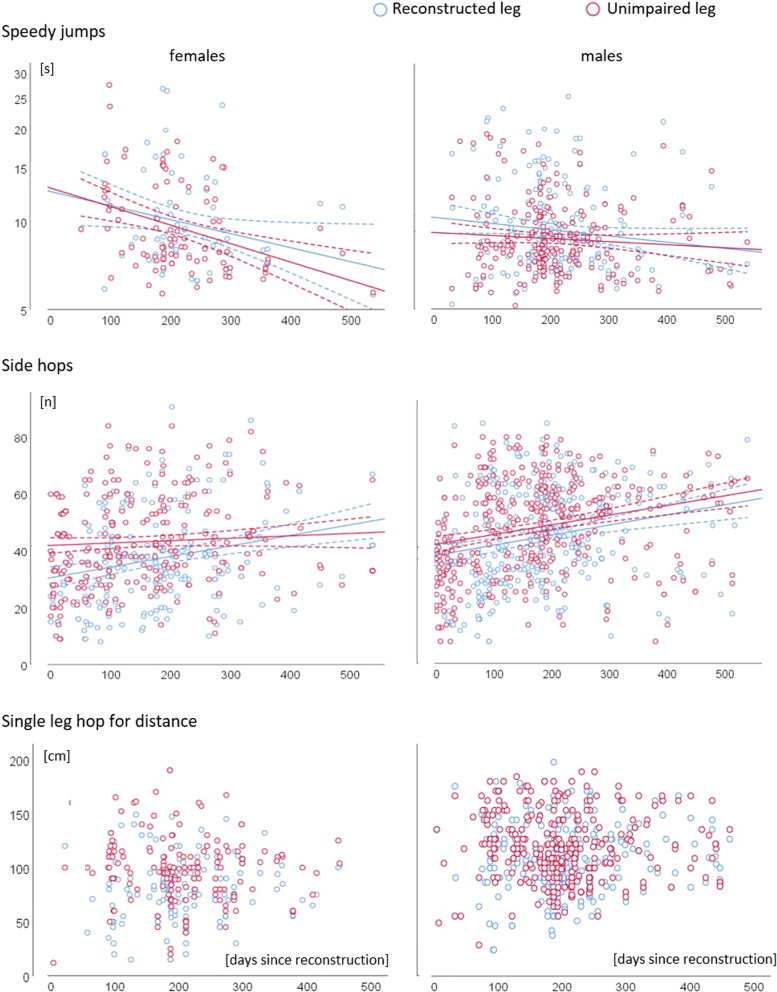
Scatterplots for the horizontal jump and hop measurements speedy jumps, side hops, and single leg hops for distance. Individual data (dots) for the days since reconstruction (x-axis) and the values of the outcomes (y-axis), separated for females and males, and the corresponding regression curves are displayed

Figure 3 displays the individual data for the horizontal jump and hop measurements (speedy jumps, side hops, and single leg hops for distance). The corresponding analysis’ outcomes are highlighted in Table 4. Mean pain intensity during measurement was 0.42 points (standard deviation 1.12 points) for the reconstructed leg only. Variance are explained by interindividual differences was 43% (speedy jumps), 14% (side hops), and 7% (single leg hops for distance).

**Table 4:** Estimates, confidence intervals, and statistical outcomes for the mixed models for horizontal jump and hop measurements speedy jumps (A), side hops (B), and single leg hops for distance (C); each time separated by the reconstructed and unimpaired

		ACL reconstructed side					Contralateral side				
		Estimate	Standard error	95% Confidence interval		p value	Estimate	Standard error	95% Confidence interval		p value
				Lower level	Upper level				Lower level	Upper level	
*A Speedy jump [seconds] n = 299 individuals, n = 846 measurements*											
	Constant	6.99	0.80	5.43	8.56	**0.001**	5.08	1.15	2.79	7.37	**0.001**
Time	time since reconstruction [days]	− 0.0006	0.0008	− 0.0021	0.0010	0.47	− 0.0009	0.0006	− 0.0020	0.0002	0.12
Individual characteristics	Being female	0.69	0.56	− 0.42	1.79	0.22	1.50	0.85	− 0.19	3.18	0.08
	Being male (ref.)	0	0				0	0			
	Age [years]	0.12	0.03	0.06	0.17	**0.001**	0.17	0.04	0.08	0.25	**0.001**
	pain intensity [a.u.]	1.34	0.34	0.67	2.01	**0.001**	0	0			
*B* *Side hops [n]* *n = 684 individuals, n = 2690 measurements*											
	Constant	54.7	2.2	50.3	59.1	**0.001**	57.9	2.5	53.0	62.7	**0.001**
Time	Time from injury to reconstruction [days]	0.002	0.001	0.000	0.003	**0.03**	− 0.0033	0.0014	− 0.0061	− 0.0006	**0.017**
	Time since reconstruction [days]	0.009	0.002	0.005	0.012	**0.001**	0.007	0.002	0.003	0.010	**0.001**
Individual characteristics	Age [years]	− 0.28	0.07	− 0.42	− 0.15	**0.001**	− 0.29	0.08	− 0.45	− 0.13	**0.001**
	Pain intensity [a.u.]	− 2.24	0.45	− 3.12	− 1.36	**0.001**	0	0			
	Being female	− 9.77	1.56	− 12.85	− 6.70	**0.001**	− 5.57	1.59	− 8.70	− 2.44	**0.001**
	Being male (ref.)	0	0				0	0			
*C Single leg hop for distance [cm] n = 465 individuals, n = 1402 measurements*											
	Constant	152.7	12.7	127.7	177.6	**0.001**	166.9	12.8	141.4	192.3	**0.001**
Time	Time since reconstruction [days]	0.056	0.026	0.004	0.107	**0.04**	0.016	0.028	− 0.039	0.072	0.6
	Time from injury to reconstruction [days]	− 0.001	0.015	− 0.031	0.028	0.9	0.005	0.015	− 0.025	0.036	0.7
Individual characteristics	Being female	− 36.6	5.3	− 47.0	− 26.2	**0.001**	− 36.5	5.8	− 48.1	− 25.0	**0.001**
	Being male (ref.)	0	0				0^c^	0			
	Age [years]	− 1.26	0.27	− 1.80	− 0.72	**0.001**	− 0.99	0.27	− 1.53	− 0.44	**0.001**
	Pain intensity [a.u.]	− 9.05	3.01	− 15.00	− 3.10	**0.003**	0	0			
Graft type	Hamstrings tendon	− 1.72	9.29	− 20.1	16.6	0.9	2.7	10.1	− 17.3	22.7	0.8
	Patellar tendon	19.8	16.1	− 11.9	51.5	0.2	67.5	31.0	5.9	129.1	**0.001**
	Quadriceps tendon (ref.)	0	0				0	0			

*N* numbers; *ACL* anterior cruciate ligament; *LL* lower level; *UL* upper level (of the 95% confidence interval); *ref.* reference

## Discussion

Function after anatomic ACL reconstruction is influenced by several interacting factors. Adopting a repeated measure cohort design, we evaluated the contribution of time between injury and surgery, time since reconstruction, age, gender, pain, graft type, and concomitant injuries as isolated and interactive contributors to inertial sensor-assessed motor function after ACL reconstructions in a multiple linear mixed model regression approach. We found that many contributors interactively impact on different functional abilities during the RTS-process after ACL reconstruction. When (all analysis performed in omnibus models) other relevant contributors are considered, the reconstructed leg function was associated with the time from injury to reconstruction (angle reproduction error, vertical hopping height, side hops) and positively with the time passed since reconstruction (vertical hopping height, side hops, single leg hop for distance). A further negative contributor to the reconstructed leg’s performance was pain intensity. Angle reproduction was worse when the lateral ligament was involved and better outcomes in the Y-Balance composite score and vertical hopping height were observed in patella grafts reconstructed knees. Most of the outcomes, independent of the leg, were also different between sexes/genders (males often showed larger function values), and affected by increasing age (negatively). The unimpaired leg was, further, mostly influenced by the time between injury and reconstruction (negatively, vertical hopping height, and side hops) and time since reconstruction (positively, Drop Jump knee displacement, vertical hopping height and side hops); but also, in parts, from the graft type of the reconstructed leg ( better outcomes in the single leg hop for distance after patella tendon graft).

Knowing these contributors, their contributive value (estimate), and their interaction is helpful for the function-based and deficit-oriented rating and management of rehabilitation and RTS strategies. Improving or even restoring functional abilities and thus decreasing the identified deficit may consequently lead to a decrease in the subsequent injury risk [7].

As the putatively unimpaired leg is affected by the injury, reconstruction, and all the measures after the injury, too, an individualized and side-dependent comparison of the function after ACL-reconstruction may be more accurate than, as it is usually done, a rating the functional status during RTS using limb symmetry indexes (LSI). The LSI approach may overestimate the knee function after ACL reconstruction [31]. This critique against the LSI is not new. Our findings, however, add a somewhat new aspect to this discussion. When the LSI and not each leg is considered, patient demographics or even intra-operative predictors do not predict the achievement of a symmetrical muscle function [32]. Our findings showed that the putatively unimpaired leg is affected by (in parts) different, but still injury-related variables, than the ACL-reconstructed leg such as graft type. This is another hint for the fact that an ACL injury is rather a global central than a local peripheral problem is given [33–35].

A function rating by estimated preinjury capacity level calculations be more constructive than LSIs-based ratings [31]. Supported by the present findings, an isolated view on the change over time of the functional abilities of the reconstructed side may be equally constructive. Despite the critique, LSIs are, nevertheless, predictive for a second ACL injury [4–6]. The comparable small effect sizes of the prediction of a second injury might, in parts, be explained by the limited value of LSIs in performance and biomechanical measures. Targeting the identified impairments in functional ability directly may reduce ACL injury risk in healthy limbs in male athletes playing level 1 sports [36].

Our findings of the relevance of the time span between injury and reconstruction is, generally in accordance with the most recent evidence. Here, the authors conclude, that “early ACLR was superior to elective delayed ACLR in terms of the Lysholm score at 2 years and the IKDC score” [37]. In contrast, it is also important to notice that most of the clinical outcomes were not different between early and late reconstruction [37]. As the measures between the ACL injury and reconstruction, like pre-operative rehabilitation affects function, they must be considered, likewise [38]. We found that the way how the time between injury and reconstruction potentially affects post-surgery functional outcomes is not equal between sides: of the unaffected leg, side hops were negatively, vertical hops positively associated with time between injury and reconstruction. A potential explanation may be found in the injury-related reduced afferent neural input from the injured side and the herewith associated globally reduced motor control output [34]. In a more complex motor control task like side hops, the limited output may be more relevant than in a somewhat simple strength/acceleration task like vertical jumps. Speculatively, this may leads to the decrease in side hops performance. Here, prehabilitation strategies may be more helpful to restore the functional ability than the potential impairment due to the lack of afferent input to the central nervous system. This assumption is highly speculative but leads to interesting experimental future study rationales. The time between injury and reconstruction is derived from many factors. Inter alia, non-coping athletes are often reconstructed before the restoration of their preinjury functional abilities [39].

Although the time passed since reconstruction was found to be a relevant predictor of function, performing one single assessment at the hypothetical end of the RTS process is not constructive as, of course and once more proved by our results, many other factors contribute to the final functional outcome [40]. Multiple repetitive measurements, aiming to monitor and verify the course of the RTS process, is more promising [40]. These repetitive measurement approach over time both considers time and (functional) status factors and was found to feasible in an athletic RTS-setting [41].

Numerous reconstruction-specific factors were also important. Previous findings of better functional outcomes after quadriceps tendon autografts, when compared with hamstrings autograft, could not be reproduced: both graft types showed comparable functional values (hamstrings even slightly better in Y-balance) [13]. Patella graft was found to be associated with better outcomes than the other graft types. When these findings are rated, one must consider that patella autografts may lead to severe reconstructed-site pain [13]. As pain was considered as independent variable in the present analyses, higher pain values during objectively equal functional tasks lead to an increase in the total model values. The impact of concomitant (meniscus and collateral ligament injuries, or even unhappy triad) injuries on the functional outcomes is, generally, in accordance with current literature [15, 16]. Reasons for this association can be found in a certain accumulation of injury- and surgery-derived functional deficits of the two (or more) injuries [42, 43].

### Limitations

The association of function and age, sex/gender pain intensity/perception during performance is not surprising, but must also be considered when function should be rated more holistic and cumulated in one model [14]. Here, the interactive calculation of the various factors in total models where the interrelationship can be operationalized (and not only single contributors to a dependent variable), must be considered as a strength of this analysis. However, we only reported associations/observations and no experimentally derived effects. That must definitely be considered as a major limitation. It is, for example, not always known why a certain measurement was undertaken at a certain time point. Furthermore, the tests themselves are (mostly) valid and reliable, the objectivity (inter-rater-reliability) of performing them in a non-laboratory clinical setting is not. Numerous further factors are known to contribute to post-reconstruction function. Here, pre-injury functional status and level of physical activity as well as the amount and type of pre- and post-surgery rehabilitation, or further functional outcomes such as strength, and more chaotic hop tests are mentioned as the (potentially) most relevant.

## Conclusion

Numerous factors such as time between injury and reconstruction, time since reconstruction, age, gender, pain, graft type, and concomitant injuries are predictive for the individual values and courses of functional abilities after ACL reconstruction. Some of the contributors to motor function such as rehabilitation measures and time until surgery can be modified. Other contributors, such as age, gender, and concomitant injuries cannot be impacted. They, however, must be considered when the post-reconstruction function is rated. It might not be enough to assess factors isolated; the knowledge on their interactive contribution to motor function is helpful for the management of the reconstruction, deficit-oriented function-based rehabilitation, and individualized return to sports strategies.

## Data Availability

All data available are presented in the manuscript.

## References

[1] Wiggins AJ, Grandhi RK, Schneider DK, Stanfield D, Webster KE, Myer GD (2016). Risk of secondary injury in younger athletes after anterior cruciate ligament reconstruction: a systematic review and meta-analysis. Am J Sports Med.

[2] Niederer D, Engeroff T, Wilke J, Vogt L, Banzer W (2018). Return to play, performance, and career duration after anterior cruciate ligament rupture: a case–control study in the five biggest football nations in Europe. Scand J Med Sci Sports.

[3] Herring SA, Kibler WB, Putukian M (2012). The team physician and the return-to-play decision: a consensus statement-2012 update. Med Sci Sports Exerc.

[4] Ashigbi EYK, Banzer W, Niederer D (2020). Return to sport tests' prognostic value for reinjury risk after anterior cruciate ligament reconstruction: a systematic review. Med Sci Sports Exerc.

[5] Grindem H, Snyder-Mackler L, Moksnes H, Engebretsen L, Risberg MA (2016). Simple decision rules can reduce reinjury risk by 84% after ACL reconstruction: the Delaware-Oslo ACL cohort study. Br J Sports Med.

[6] Kyritsis P, Bahr R, Landreau P, Miladi R, Witvrouw E (2016). Likelihood of ACL graft rupture: not meeting six clinical discharge criteria before return to sport is associated with a four times greater risk of rupture. Br J Sports Med.

[7] Hewett TE, Di Stasi SL, Myer GD (2013). Current concepts for injury prevention in athletes after anterior cruciate ligament reconstruction. Am J Sports Med.

[8] van Melick N, van Cingel REH, Brooijmans F, Neeter C, van Tienen T, Hullegie W, Nijhuis-van der Sanden MWG (2016). Evidence-based clinical practice update: practice guidelines for anterior cruciate ligament rehabilitation based on a systematic review and multidisciplinary consensus. Br J Sports Med.

[9] Meredith SJ, Rauer T, Chmielewski TL, Fink C, Diermeier T, Rothrauff BB (2020). Return to sport after anterior cruciate ligament injury: panther symposium ACL injury return to sport consensus group. Orthop J Sports Med.

[10] Nagelli CV, Hewett TE (2017). Should return to sport be delayed until 2 years after anterior cruciate ligament reconstruction?. Biol Funct Consid Sports Med.

[11] Ebert JR, Webster KE, Edwards PK, Joss BK, D'Alessandro P, Janes G, Annear P (2019). Current perspectives of Australian therapists on rehabilitation and return to sport after anterior cruciate ligament reconstruction: a survey. Phys Ther Sport.

[12] Grassi A, Carulli C, Innocenti M, Mosca M, Zaffagnini S, Bait C (2018). New trends in anterior cruciate ligament reconstruction: a systematic review of national surveys of the last 5 years. Joints.

[13] Mouarbes D, Menetrey J, Marot V, Courtot L, Berard E, Cavaignac E (2019). Anterior cruciate ligament reconstruction: a systematic review and meta-analysis of outcomes for quadriceps tendon autograft versus bone-patellar tendon-bone and hamstring-tendon autografts. Am J Sports Med.

[14] Leister I, Mattiassich G, Kindermann H, Ortmaier R, Barthofer J, Vasvary I (2018). Reference values for fatigued versus non-fatigued limb symmetry index measured by a newly designed single-leg hop test battery in healthy subjects: a pilot study. Sport Sci Health.

[15] Pedersen M, Johnson JL, Grindem H, Magnusson K, Snyder-Mackler L, Risberg MA (2020). Meniscus or cartilage injury at the time of anterior cruciate ligament tear is associated with worse prognosis for patient-reported outcome 2 to 10 years after anterior cruciate ligament injury: a systematic review. J Orthop Sports Phys Ther.

[16] Everhart JS, DiBartola AC, Swank K, Pettit R, Hughes L, Lewis C, Flanigan DC (2020). Cartilage damage at the time of anterior cruciate ligament reconstruction is associated with weaker quadriceps function and lower risk of future ACL injury. Knee Surg Sports Traumatol Arthrosc.

[17] Davies GJ (2017). Individualizing the return to sports after anterior cruciate ligament reconstruction. Oper Tech Orthop.

[18] Wilk KE, Arrigo CA (2017). Rehabilitation principles of the anterior cruciate ligament reconstructed knee: twelve steps for successful progression and return to play. Clin Sports Med.

[19] Taberner M, Allen T, Cohen DD (2019). Progressing rehabilitation after injury: consider the 'control-chaos continuum'. Br J Sports Med.

[20] Bronner S, Agraharasamakulam S, Ojofeitimi S (2010). Reliability and validity of electrogoniometry measurement of lower extremity movement. J Med Eng Technol.

[21] Olsson L, Lund H, Henriksen M, Rogind H, Bliddal H, Danneskiold-Samsøe B (2004). Test–retest reliability of a knee joint position sense measurement method in sitting and prone position. Adv Physiother.

[22] Shaffer SW, Teyhen DS, Lorenson CL, Warren RL, Koreerat CM, Straseske CA, Childs JD (2013). Y-balance test: a reliability study involving multiple raters. Mil Med.

[23] Ford KR, Myer GD, Hewett TE (2007). Reliability of landing 3D motion analysis: implications for longitudinal analyses. Med Sci Sports Exerc.

[24] Gustavsson A, Neeter C, Thomeé P, Silbernagel KG, Augustsson J, Thomeé R, Karlsson J (2006). A test battery for evaluating hop performance in patients with an ACL injury and patients who have undergone ACL reconstruction. Knee Surg Sports Traumatol Arthrosc.

[25] Wang J, Xu J, Shull PB (2018). Vertical jump height estimation algorithm based on takeoff and landing identification via foot-worn inertial sensing. J Biomech Eng.

[26] Hildebrandt C, Müller L, Zisch B, Huber R, Fink C, Raschner C (2015). Functional assessments for decision-making regarding return to sports following ACL reconstruction. Part I: development of a new test battery. Knee Surg Sports Traumatol Arthrosc.

[27] Keller M, Kurz E, Schmidtlein O, Welsch G, Anders C (2016). Interdisciplinary assessment criteria for rehabilitation after injuries of the lower extremity: a function-based return to activity algorithm. Sportverletz Sportschaden.

[28] Reid A, Birmingham TB, Stratford PW, Alcock GK, Giffin JR (2007). Hop testing provides a reliable and valid outcome measure during rehabilitation after anterior cruciate ligament reconstruction. Phys Ther.

[29] Dan MJ, Lun KK, Dan L, Efird J, Pelletier M, Broe D, Walsh WR (2019). Wearable inertial sensors and pressure MAT detect risk factors associated with ACL graft failure that are not possible with traditional return to sport assessments. BMJ Open Sport Exerc Med.

[30] Mitternacht J, Hermann A, Carqueville P (2022). Acquisition of lower-limb motion characteristics with a single inertial measurement unit—validation for use in physiotherapy. Diagnostics.

[31] Wellsandt E, Failla MJ, Snyder-Mackler L (2017). Limb symmetry indexes can overestimate knee function after anterior cruciate ligament injury. J Orthop Sports Phys Ther.

[32] Hamrin Senorski E, Svantesson E, Beischer S, Thomeé C, Grassi A, Krupic F (2018). Concomitant injuries may not reduce the likelihood of achieving symmetrical muscle function one year after anterior cruciate ligament reconstruction: a prospective observational study based on 263 patients. Knee Surg Sports Traumatol Arthrosc.

[33] Swanik CB (2015). Brains and sprains: the brain's role in noncontact anterior cruciate ligament injuries. J Athl Train.

[34] Grooms D, Appelbaum G, Onate J (2015). Neuroplasticity following anterior cruciate ligament injury: a framework for visual-motor training approaches in rehabilitation. J Orthop Sports Phys Ther.

[35] Zarzycki R, Morton SM, Charalambous CC, Pietrosimone B, Williams GN, Snyder-Mackler L (2020). Athletes after anterior cruciate ligament reconstruction demonstrate asymmetric intracortical facilitation early after surgery. J Orthop Res.

[36] King E, Richter C, Daniels KAJ, Franklyn-Miller A, Falvey E, Myer GD (2021). Can biomechanical testing after anterior cruciate ligament reconstruction identify athletes at risk for subsequent ACL injury to the contralateral uninjured limb?. Am J Sports Med..

[37] Shen X, Liu T, Xu S, Chen B, Tang X, Xiao J, Qin Y (2022). Optimal timing of anterior cruciate ligament reconstruction in patients with anterior cruciate ligament tear: a systematic review and meta-analysis. JAMA Netw Open.

[38] Giesche F, Niederer D, Banzer W, Vogt L (2020). Evidence for the effects of prehabilitation before ACL-reconstruction on return to sport-related and self-reported knee function: a systematic review. PLoS One.

[39] Hartigan EH, Axe MJ, Snyder-Mackler L (2010). Time line for noncopers to pass return-to-sports criteria after anterior cruciate ligament reconstruction. J Orthop Sports Phys Ther.

[40] Dingenen B, Gokeler A (2017). Optimization of the return-to-sport paradigm after anterior cruciate ligament reconstruction: a critical step back to move forward. Sports Med.

[41] Niederer D, Wilke J, Krause F, Banzer W, Engeroff T (2019). Integrating the evidence and clinical expertise in the shared decision and graduated return to sport process: a time series case study after anterior cruciate ligament rupture and reconstruction. J Orthop Case Rep.

[42] Eitzen I, Grindem H, Nilstad A, Moksnes H, Risberg MA (2016). Quantifying quadriceps muscle strength in patients with ACL injury, focal cartilage lesions, and degenerative meniscus tears: differences and clinical implications. Orthop J Sports Med.

[43] Løken S, Ludvigsen TC, Høysveen T, Holm I, Engebretsen L, Reinholt FP (2009). Autologous chondrocyte implantation to repair knee cartilage injury: ultrastructural evaluation at 2 years and long-term follow-up including muscle strength measurements. Knee Surg Sports Traumatol Arthrosc.

